# Cloning, functional expression and characterization of a bifunctional 3-hydroxybutanal dehydrogenase /reductase involved in acetone metabolism by *Desulfococcus biacutus*

**DOI:** 10.1186/s12866-016-0899-9

**Published:** 2016-11-25

**Authors:** Jasmin Frey, Hendrik Rusche, Bernhard Schink, David Schleheck

**Affiliations:** Department of Biology, University of Konstanz, Postbox 649, D-78457 Konstanz, Germany

**Keywords:** Acetone activation, Sulfate-reducing bacteria, Carbonylation, Bifunctional MDR superfamily oxidoreductase

## Abstract

**Background:**

The strictly anaerobic, sulfate-reducing bacterium *Desulfococcus biacutus* can utilize acetone as sole carbon and energy source for growth. Whereas in aerobic and nitrate-reducing bacteria acetone is activated by carboxylation with CO_2_ to acetoacetate, *D. biacutus* involves CO as a cosubstrate for acetone activation through a different, so far unknown pathway. Proteomic studies indicated that, among others, a predicted medium-chain dehydrogenase/reductase (MDR) superfamily, zinc-dependent alcohol dehydrogenase (locus tag DebiaDRAFT_04514) is specifically and highly produced during growth with acetone.

**Results:**

The MDR gene DebiaDRAFT_04514 was cloned and overexpressed in *E. coli*. The purified recombinant protein required zinc as cofactor, and accepted NADH/NAD^+^ but not NADPH/NADP^+^ as electron donor/acceptor. The pH optimum was at pH 8, and the temperature optimum at 45 °C. Highest specific activities were observed for reduction of C_3_ - C_5_-aldehydes with NADH, such as propanal to propanol (380 ± 15 mU mg^−1^ protein), butanal to butanol (300 ± 24 mU mg^−1^), and 3-hydroxybutanal to 1,3-butanediol (248 ± 60 mU mg^−1^), however, the enzyme also oxidized 3-hydroxybutanal with NAD^+^ to acetoacetaldehyde (83 ± 18 mU mg^−1^).

**Conclusion:**

The enzyme might play a key role in acetone degradation by *D. biacutus*, for example as a bifunctional 3-hydroxybutanal dehydrogenase/reductase. Its recombinant production may represent an important step in the elucidation of the complete degradation pathway.

**Electronic supplementary material:**

The online version of this article (doi:10.1186/s12866-016-0899-9) contains supplementary material, which is available to authorized users.

## Background

*Desulfococcus biacutus* strain KMRActS is a Gram-negative, sulfate-reducing deltaproteobacterium capable of using acetone as sole carbon and electron source [1]. Due to the small energy budget of this bacterium, activation of acetone by carboxylation to acetoacetate with concomitant hydrolysis of two (or more) ATP equivalents, as found in aerobic and nitrate-reducing bacteria [2–6], is hardly possible. Early physiological findings indicated that acetoacetate is not a free intermediate in the degradation pathway [7]. Correspondingly, no acetone carboxylase activity was detected in cell-free extracts, and no acetone carboxylases were found in the genome and proteome of this bacterium [7–9]. Experiments with dense cell suspensions and cell-free extracts suggested that acetone may be activated through a carbonylation or a formylation reaction, and that acetoacetaldehyde rather than acetoacetate may be formed as an intermediate [3, 8]. In cell-free extracts of acetone-grown *D. biacutus* cells, acetoacetaldehyde was trapped as its dinitrophenylhydrazone derivative and was identified by mass spectrometry, after reactions with acetone, ATP and CO as cosubstrates [8]. This reaction was not observed in cell-free extract of butyrate-grown cells, hence, the proposed acetone-activating enzyme, and most likely the entire acetone utilization pathway, is inducibly expressed in *D. biacutus.* Nonetheless, the mechanism of the acetone activation reaction remains unknown so far.

A differential-proteomics approach comparing acetone- and butyrate-grown *D. biacutus* cells revealed several proteins/genes that were specifically and strongly induced in acetone-grown cells, but not in butyrate-grown cells [9]. One of the most prominent acetone-inducible proteins observed is encoded by gene (IMG locus tag) DebiaDRAFT_04514, and is annotated as medium-chain dehydrogenase/reductase (MDR) superfamily alcohol dehydrogenase (COG1063 in the Clusters of Orthologous Groups classification system). Other strongly induced proteins are a predicted thiamine diphosphate (TDP)-requiring enzyme (COG0028), and a cobalamin (B_12_)-binding subunit (COG2185) of a methylmalonyl-CoA mutase-like complex [9].

Alcohol dehydrogenases (ADH) usually catalyze the reversible oxidation of primary or secondary alcohols to aldehydes or ketones, and the reactions are coupled to the reduction/oxidation of a pyridine nucleotide [10, 11]. Further, there are three types of ADHs known which are classified by the absence or presence, and the type of incorporated metal ion: ADHs that are independent of a metal ion, iron-dependent ADHs, which can be oxygen-sensitive [12, 13], and zinc-dependent ADHs; DebiaDRAFT_04514 is predicted as a zinc-dependent ADH.

In the present study, we cloned, heterologously expressed, purified, and characterized the acetone-inducible gene/protein DebiaDRAFT_04514, in an attempt to gain a better understanding of its possible role in the acetone utilization pathway of *D. biacutus*. This is also the first description of a functionally expressed recombinant enzyme originating from this bacterium.

## Methods

### Chemicals

All chemicals were at least of analytical grade and were purchased from Sigma-Aldrich (Germany), Carl Roth GmbH (Germany) or Merck KGaA (Germany). Biochemicals (NADH, NADPH, NAD^+^ and NADP^+^) were purchased from Sigma-Aldrich (Germany). 3-Hydroxybutanal was synthesized by Dr. Thomas Huhn and Fabian Schneider, Chemistry Department of University of Konstanz.

### Bacterial growth conditions

*Desulfococcus biacutus* strain KMRActS (DSM5651) was grown in sulfide-reduced, CO_2_/bicarbonate-buffered (pH 7.2), freshwater mineral-salts medium as described previously [7, 8]. The medium was supplemented with 5 mM acetone as sole carbon and energy source, and with 10 mM sulfate as electron acceptor. Cultures were incubated at 30 °C in the dark under strictly anoxic conditions. *Escherichia coli* strains TOP10 (Invitrogen) and Rosetta 2 (Merck) were grown aerobically (shaking) in lysogenic broth (LB) medium (10 g l^-1^ peptone, 5 g l^−1^ yeast extract, 10 g l^−1^ NaCl) supplemented with 100 μg ml^−1^ ampicillin.

### Plasmid construction and overexpression

The Qiagen Genomic DNA Kit (Qiagen, Germany) was used for preparation of genomic DNA of *D. biacutus*. A cell pellet obtained from a 50-ml culture with OD_600_ ~ 0.3 was resuspended in 1 ml of sterile, DNA-free H_2_O, and further processed following the manufacturer’s protocol. For construction of expression plasmids, the Champion™ pET Directional TOPO® Expression Kit (Invitrogen) was used (N-terminal His_6_-tag). The gene of interest of *D. biacutus* was amplified by PCR, using the forward primer 5′-CACCATGGCAAAAATGATGAAAACAT-3′ (TOPO-cloning overhang underlined) and reverse primer 5′-AACAAAAAAACACTCGACTACATA-3′; the PCR polymerase used was Phusion® High-Fidelity DNA Polymerase (New England Biolabs), and PCR conditions were 35 cycles of 45s denaturation at 98 °C, 45s annealing at 60 °C, and 90s elongation at 72 °C. The PCR product was ligated into the expression vector pET100 (Invitrogen), and cloning was performed as recommended by the manufacturer. Plasmid DNA of positive clones was purified using Zyppy Plasmid Miniprep Kit (Zymo Research, Germany), and correct integration of the insert was confirmed by sequencing (GATC-Biotech, Constance, Germany). The DNASTAR Lasergene 5 software package was used for primer design and for sequence data analysis.

Purified plasmid DNA was used for transformation of chemically competent *E. coli* Rosetta 2 (DE3) cells (Merck KGaA, Germany). Cells were grown in LB medium (containing 100 μg ml^−1^ ampicillin and 35 μg ml^−1^ chloramphenicol) at 37 ° C to OD_600_ 0.4–0.8, followed by addition of 0.5 mM isopropyl β-D-1-thiogalactopyranoside (IPTG) and 3% (*v/v*) of ethanol. After induction, the cultures were incubated for further 5h at 18 °C, and then harvested by centrifugation at 2500 × *g* for 10 min at 4 °C.

### Preparation of cell-free extracts

*E. coli* cells were washed twice with a 20 mM Tris/HCl buffer, pH 7.2, containing 100 mM KCl and 10% (*v/v*) glycerol, and resuspended in the same buffer supplemented with 0.5 mg ml^−1^ DNase and 1 mg ml^−1^ protease inhibitor (Complete Mini, EDTA-free protease inhibitor cocktail tablets, Roche Diagnostics GmbH, Germany) prior to disruption by three passages through a cooled French pressure cell (140 MPa). Cell debris and intact cells were removed by centrifugation (16,000 × *g*, 10min, 4 °C), and the soluble protein fraction was separated from the membrane protein fraction by ultracentrifugation (104,000 × *g*, 1h, 4 °C). Cell-free extract of *D. biacutus* was prepared as described before [8].

### Purification of His-tagged proteins

The supernatant containing the soluble protein fraction obtained by ultracentrifugation was loaded on a Protino Ni-NTA column (Macherey-Nagel, Germany) pre-equilibrated with buffer (20 mM Tris/HCl, pH 7.2, 100 mM KCl, 10% (*v/v*) glycerol). Unspecifically bound proteins were washed off stepwise with the buffer described above containing 20 and 40 mM imidazole. The bound His-tagged proteins were eluted with the same buffer containing 250 mM imidazole. Eluted proteins were concentrated with an Amicon Ultra-15 Centrifugal Filter Device (10 kDa cutoff; Merck Millipore) while the buffer was exchanged twice against the same buffer containing 50 μM ZnCl_2_. After addition of 30% (*v/v*) glycerol, the purified, concentrated proteins were stored in aliquots at -20 °C. Protein concentrations were determined after Bradford with bovine serum albumin (BSA) as standard [14].

### Protein gel electrophoresis and identification

For analysis of expression and purification of recombinant protein, one-dimensional denaturing polyacrylamide gel electrophoresis (SDS-PAGE) was performed with a 4% stacking gel and a 12% resolving gel [15], and with PageRuler Prestained Protein Ladder (Thermo Scientific) as a reference; gels were run at a constant current of 20 mA per gel for 1.5h. For an estimation of the size of the enzyme complex, native PAGE was performed using Mini-Protean TGX Precast Gels (Bio-Rad) with a polyacrylamide gradient of 4 – 15%; Amersham High Molecular Weight Calibration Kit (GE Healthcare) was used as a reference, and gels were run with native-gel running buffer (192 mM Glycine, 25 mM Tris/HCl pH 8.8; without SDS) under constant current of 8 mA per gel for 3h [15, 16]. Protein staining was performed by colloidal Coomassie staining with final concentrations 2% H_3_PO_4_, 10% (NH_4_)_2_SO_4_, 20% methanol, and 0.08% (*w/v*) Coomassie Brilliant Blue R-250 [17]. Protein bands excised from gels or soluble proteins in preparations were identified by peptide fingerprinting mass spectrometry at the Proteomics facility of University of Konstanz, as described previously [9].

### Enzyme assays

All enzyme assays were performed routinely under anoxic conditions, i.e., under N_2_ gas in cuvettes with rubber stoppers, either in 25 mM MOPS (3-(*N*-morpholino)propanesulfonic acid) buffer (pH 6.0, 7.2 or 8.0) containing 1 g l^−1^ NaCl, 0.6 g l^−1^ MgCl_2_ × 6 H_2_O, or in 50 mM Tris/HCl buffer (pH 9.0), each containing 3 mM DTT and 50 μM ZnCl_2_. Reduction of substrates was carried out with 0.1 mM NADH (or NADPH), and oxidation of substrates was performed with 0.5 or 2.5 mM NAD^+^ (or NADP^+^), as co-substrates, as specified in Table 1. Reactions were started by addition of 5 mM substrate followed by spectrophotometrical measurement of absorption (increase or decrease) of NADH at 340 nm (ε_NADH_ = 6.292 mM^-1^ • cm^−1^) [18].

**Table 1:** Specific NAD(H)-dependent oxidoreductase activities determined for the heterologously expressed and purified Debia-MDR protein

Reduction with NADH			Oxidation with NAD^+^		
Substrate^1)^		Spec. activity mU mg^−1^	Substrate^2)^		Spec. activity mU mg^−1^
	Formaldehyde	b.d.		Methanol	n.d.
	Acetaldehyde	52 ± 14		Ethanol	73 ± 13
	Propanal	380 ± 15		1-Propanol^b^	22 ± 2
	Butanal	301 ± 24		1-Butanol	47 ± 15
	Isobutanal	276 ± 30		Isobutanol	n.d.
	Pentanal	325 ± 35		1-Pentanol	11 ± 3
	Benzaldehyde	b.d.		Benzyl alcohol	n.d.
	Propanone (Acetone)	93 ± 2		2-Propanol^a^ (Isopropanol)	21 ± 1
	Butanone	65 ± 11		2-Butanol^b^	115 ± 8
	2-Pentanone	126 ± 38		2-Pentanol	n.d.
	3-Pentanone	141 ± 19		3-Pentanol	n.d.
	2-Hexanone	45 ± 9		2-Hexanol	n.d.
	3-Hydroxybutanone (Acetoine)	326 ± 38		2,3-Butanediol	150 ± 8
	2,3-Butandione (Diacetyl)	298 ± 42		3-Hydroxybutanone (Acetoine)	b.d.
	3-Hydroxybutanal	248 ± 59		1,3-Butanediol	80 ± 23
	4-Hydroxy-2-butanone	155 ± 31			
	3-Oxobutanal (Acetoacetaldehyde)	n.s.		3-Hydroxybutanal	83 ± 18
				4-Hydroxy-2-butanone	18 ± 3

*b.d.* below detection limit (<1 mU mg^−1^ protein), *n.d.* not determined, *n.s.* no substrate was available for testing

^1)^Assay conditions: anoxic 25 mM MOPS buffer (pH 7.2) plus 3 mM DTT and 50 μM ZnCl_2_, 30 °C. Reactions in reductive direction were assayed with 0.1 mM NADH. Reactions were started by addition of 5 mM substrate

^2)^Assay conditions: anoxic 25 mM MOPS buffer (pH 8.0) plus 3 mM DTT and 50 μM ZnCl_2_, 30 °C. Reactions in the oxidative direction were assayed with 2.5 mM NAD^+^, or at pH 7.2 with 0.5 mM NAD^+^(^a^), or at pH 7.2 with 2.5 mM NAD^+^(^b^)

## Results

### Predicted features of DebiaDRAFT_04514 based on its amino acid sequence

Locus tag DebiaDRAFT_04514 was predicted (IMG annotation) to encode a threonine dehydrogenase or related Zn-dependent dehydrogenase, which belongs to the MDR superfamily of alcohol dehydrogenases: DebiaDRAFT_04514 (in the following abbreviated as Debia-MDR) harbors conserved zinc-binding catalytic domains of alcohol dehydrogenases (protein domains Adh_N, ADH_zinc-N) with a GroES-like structure and a NAD(P)-binding Rossman fold. The predicted molecular mass of the Debia-MDR monomer is 38,272 Da. The MDR-family proteins in bacteria and yeasts typically form tetramers [19], and also for Debia-MDR, a tetramer interface (conserved domain cd08285) was predicted [20] (see below).

While amino acid sequence identities of different MDR family enzymes can be only 20% or less [21], Debia-MDR exhibited up to 70% sequence identity to predicted, uncharacterized alcohol dehydrogenases, e.g., of *Desulfonatronovibrio magnus* (WP_045216775) and *Geobacter uraniireducens* (ABQ28495 and WP_041246222), and 47% sequence identity to a characterized alcohol dehydrogenase of *C. beijerinckii* NRRL B-593 (locus ADH_CLOBE; P25984), which utilizes acetone and butanal as substrates [22]. In addition, Debia-MDR showed 21% sequence identity to a characterized acetoin reductase/2,3-butanediol dehydrogenase of *Clostridium beijerinckii* [23].

### Heterologous overproduction and purification of Debia-MDR

Recombinant expression of Debia-MDR with high yield was obtained with *E. coli* Rosetta 2 cells harboring the expression plasmid pET100-Debia_04514N when grown in LB medium at 37 °C to an optical density of ~ 0.5: subsequently, cells were induced by addition of isopropyl β-D-1-thiogalactopyranoside (IPTG; 0.5 mM), and upon induction, the medium was supplemented also with 3% (*v/v*) ethanol; addition of ethanol induces the heat-shock response and increases the production of chaperones (GroES/EL and DnaK/J) with positive effects on correct protein folding [24]. After induction, the cultures were incubated further for 5h at 18 °C.

**Fig. 1:**
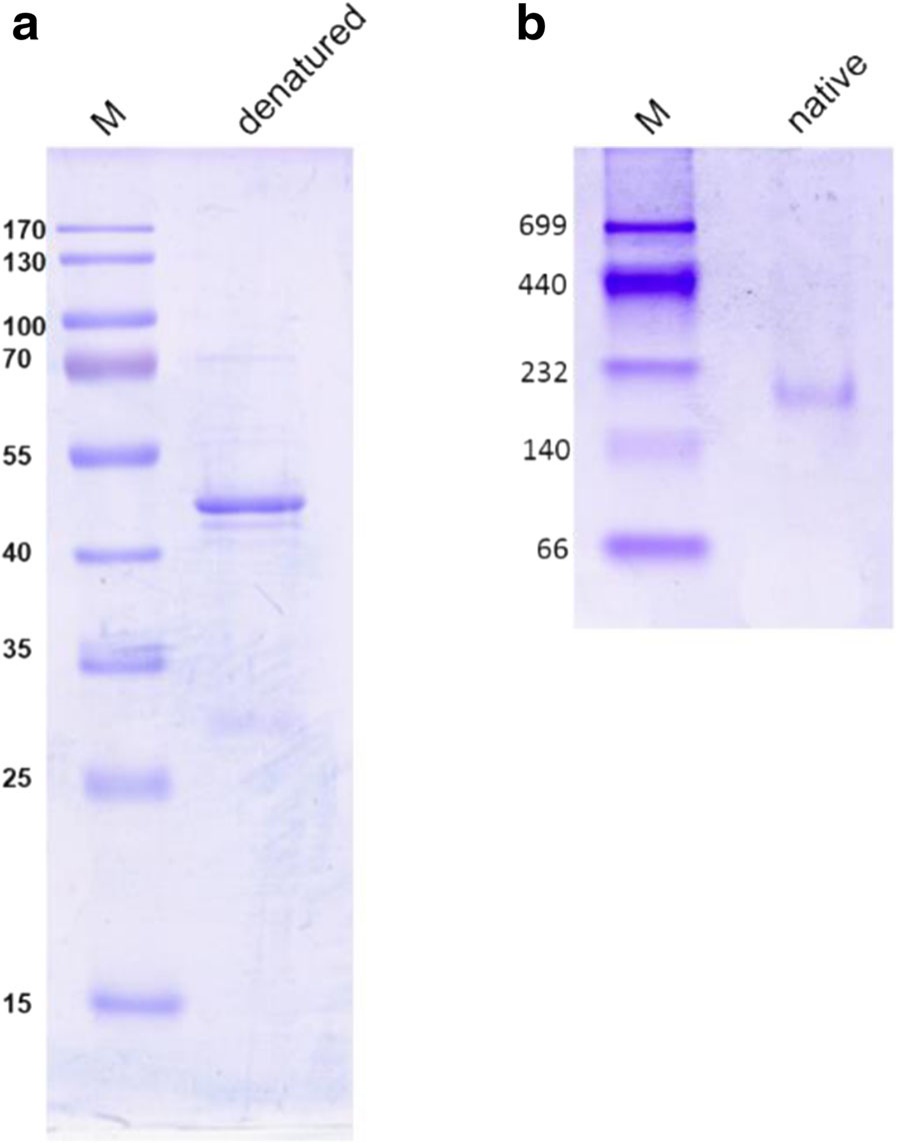
**a**, **b**. Evaluation of the purity of recombinant, His-tagged *Desulfococcus biacutus* MDR protein by denaturing PAGE (**a**), and analysis of its native molecular weight by native PAGE (**b**). M, molecular weight markers (kDa)

Figure 1a shows a representative preparation of His-tag purified protein separated on a denaturing SDS-PAGE gel. There was one major band with the expected molecular mass of approx. 41 kDa (native Debia-MDR, app. 38.3 kDa; plus His-Tag, app. 3 kDa). The identity of recombinant Debia-MDR was confirmed by peptide fingerprinting mass spectrometry. Minor contaminations in the protein preparation (see Fig. 1a) were identified as *E. coli* proteins, but not any other oxidoreductase (data not shown). In addition, also a native PAGE was performed in order to estimate the size of the native protein complex (Fig. 1b). Here, purified Debia-MDR appeared as a single protein band at about 170 kDa molecular mass. Thus, native Debia-MDR has most likely a homotetrameric structure, which is in accordance with the bioinformatic prediction of a tetramer-binding domain in its amino acid sequence (see above).

### Zinc- and NADH-dependency of Debia-MDR

A zinc-dependency of the Debia-MDR enzyme activity, as predicted by homology, was confirmed. For example, with acetone and NADH as substrates (see below), no activity was detectable in the absence of Zn^2+^. Incubation of the enzyme preparation with 50 μM Zn^2+^ for 30min prior to the enzyme assays led to a specific activity of 4.0 mU mg^-1^ protein, while addition of 50 μM Zn^2+^ to the enzyme preparation directly after the His-tag purification step (and its presence during storage at -20 °C) led to a maximal activity of 93 mU mg^−1^, each with acetone and NADH as substrates. Further, the enzyme in the presence of Zn^2+^ was inhibited completely by addition of 100 μM HgCl_2_ as typical of Zn-dependent MDR dehydrogenases, e.g., threonine 3-dehydrogenase of *E. coli*, 3-hydoxyisobutyrate dehydrogenase of *P. putida*, or a dehydrogenase of *Geotrichum capitatum* [25–27]. Further, the enzyme accepted only NAD^+^/NADH as electron acceptor/donor. No activity was detectable with NADP^+^/NADPH, which is also typical of most MDR superfamily dehydrogenase enzymes [28].

### Substrate range

The Debia-MDR enzyme showed no reaction with L-threonine as substrate and NAD^+^, as opposed to its initial sequence-based functional prediction. However, the enzyme exhibited activity with a range of short- and medium-chain aldehydes, ketones, and alcohols, as illustrated in Table 1. With respect to the aldehydes tested (Table 1), the enzyme showed highest specific activity in the reduction of propanal (380 mU mg^−1^ = 100%), followed by pentanal (85%), butanal (79%), isobutanal (72%), 3-hydroxybutanal (65%), and acetaldehyde (14%); no activity was detectable with formaldehyde or benzaldehyde. With respect to the non-substituted ketones tested (Table 1), activities were generally lower than those with aldehydes: the enzyme showed highest activity for reduction of 3-pentanone (141 mU mg^−1^ = 37% of activity with propanal), followed by 2-pentanone (33%), propanone (acetone) (24%), butanone (17%), and 2-hexanone (12%). The affinity (K_m_) of the enzyme for acetone as substrate was determined to be 0.04 mM (Additional file 1: Figure S1), which is low compared to other acetone-reducing dehydrogenases, e.g., that of *Sulfolobus solfataricus* (K_m_ = 6.6 mM) and *Equus caballus* (K_m_ = 135 mM) [11, 29]. Further, the enzyme exhibited high activity for reduction of substituted ketones, such as 3-hydroxybutanone (acetoin) (86%), 2,3-butanedione (diacetyl) (78%), and 4-hydroxy-2-butanone (41%). Unfortunately, no substrate was available to test a reduction of 3-oxobutanal (acetoacetaldehyde) (see below). The activities for the corresponding reverse reactions with NAD^+^ were lower, for example with propanol, butanol, or pentanol as substrates (<18% relative to the corresponding forward reactions with NADH), or with 2,3-butanediol (46%) or 1,3-butanediol (32%); exceptions were ethanol (140%) and 2-butanol (177%), while no activity was detectable with 3-hydroxybutanone (acetoin) (Table 1).

Interestingly, we observed that the enzyme catalyzed also the oxidation of 3-hydroxybutanal with NAD^+^, hence, a reaction in addition to the corresponding reductive reaction of the same substrate with NADH, though with lower apparent activity (app. 33% of the activity in reductive direction). Thus, the Debia-MDR appeared to be a bifunctional 3-hydroxybutanal reductase/dehydrogenase (see Discussion). A similar bi-functionality of the enzyme was confirmed with 4-hydroxy-2-butanone as substrate (see Table 1).

### pH and temperature optimum

The effect of pH on the Debia-MDR activity was tested in reactions with butanal and NADH as substrates, at pH 6.0, 7.2, 8.0 (each in 25 mM MOPS buffer) and at pH 9.0 (in 50 mM Tris/HCl buffer). The pH optimum was between pH 7.0 and 8.0 (Additional file 1: Table S1). Further, the effect of temperature on the activity was tested in the range of 25 °C to 50 °C, with butanal and NADH in MOPS buffer, pH 7.2, as described above. The highest specific activity was detected at 45 °C (Additional file 1: Table S2).

### Acetone-inducible butanal dehydrogenase / 3-hydroxybutanal reductase activity confirmed in cell-free extracts of *D. biacutus*

Enzyme assays were performed also with cell-free extracts of *D. biacutus*, in order to confirm that the reductase/dehydrogenase activity attributed to Debia-MDR is induced by acetone, as indicated already by the proteomics data [9]. For example, with butanal as substrate and NAD^+^, cell-free extract of acetone-grown cells exhibited an activity of 20 ± 3 mU mg^−1^ protein, whereas in extracts of butyrate-grown cells, the activity was 10-fold reduced (2 ± 0.1 mU mg^−1^ protein). Also with 3-hydroxybutanal as substrate in the reductive direction with NADH, the activity was ca. 3-fold higher in extracts of acetone-grown cells (7.4 ± 0.4 mU mg^−1^ protein) compared to that of butyrate-grown cells (2.6 ± 0.9 mU mg^−1^ protein).

## Discussion

Debia-MDR of *Desulfococcus biacutus*, which was found previously to be inducibly expressed during growth with acetone [9], was successfully cloned and overexpressed in *E. coli*. The features determined with the recombinant enzyme correspond well to those predicted from its amino acid sequence. The enzyme is active only in the presence of zinc, and with NAD^+^/NADH as electron acceptor/donor, but not with NADP^+^/NADPH. Further, the activity of Debia-MDR, as prepared in this study, was not sensitive to molecular oxygen, in contrast to iron-dependent dehydrogenases, which commonly are inactivated quickly under oxic conditions (half-life of minutes to a couple of hours under oxic conditions; [12, 30]). The native enzyme showed a molecular mass of app. 170 kDa, which corresponds well to its bioinformatically predicted homotetrameric structure. Other described zinc-dependent ADHs in bacteria and yeasts also exhibit a homotetrameric structure, while dimeric ADHs can be found in higher plants and mammals [31–33]. The enzyme exhibited a pH optimum between pH 7 and 8, and a slightly elevated temperature optimum (app. 45 °C); *D. biacutus* grows optimally at 30 °C and cannot grow at higher temperatures [1]. The enzyme showed reductase activity with aldehydes and ketones, preferably of a chain length of three to five carbon atoms, as far as tested in this study (Table 1). Moreover, based on the specific activities observed, aldehydes were preferred over ketones. A branched-chain aldehyde was also accepted (isobutanal), with an activity comparable to that with the linear analogue (butanal). The specific activities for alcohol oxidations determined were only about one fifth to one tenth of those for the respective reduction of aldehydes/ketones; these low activities are partly due to the unfavorable equilibria of these reactions, which typically are on the side of the alcohols.

Based on the observed substrate range and catalytic efficiencies (Table 1), and in the context of the yet limited information that is available on the acetone degradation pathway of *D. biacutus*, several roles of Debia-MDR are to be considered.

First, the enzyme could play a role in the oxidation of isopropanol to acetone, since *D. biacutus* is able to utilize also isopropanol via the acetone pathway: our preliminary proteomic analyses of isopropanol-grown cells in comparison to acetone-grown cells (data not shown) indicated that the same set of enzymes is expressed, e.g., the predicted thiamine diphosphate (TDP)-requiring enzyme (DebiaDRAFT_04566), the cobalamin (B_12_)-binding subunit of a methylmalonyl-CoA mutase-like complex (DebiaDRAFT_04573-74), and the zinc-dependent MDR described in this study (DEBIADraft_04514). However, during growth with isopropanol, yet another dehydrogenase candidate (DebiaDRAFT_04392) appeared to be additionally, and strongly produced in comparison to acetone-grown cells, and this candidate is predicted as iron-dependent alcohol dehydrogenase. Therefore, we suggest that this dehydrogenase, DebiaDRAFT_04392, may be the dehydrogenase that funnels isopropanol into the acetone pathway, and not the zinc-dependent dehydrogenase examined in this study.

Second, Debia-MDR may have a detoxifying function, by reducing overproduced toxic aldehydes, which may be formed in the acetone activation pathway, to less toxic alcohols. For example, one proposed pathway [9] that could involve the predicted TDP-requiring enzyme (DebiaDRAFT_04566) and the B_12_-binding methylmalonyl-CoA mutase-like complex (DebiaDRAFT_04573-74) may proceed via a carbonylation (or formylation) of acetone to 2-hydroxy-2-methylpropanal (2-hydroxyisobutanal) followed by linearization of this branched-chain aldehyde to 3-hydroxybutanal, respectively. Hence, Debia-MDR might play a role in a reversible conversion of, e.g., 3-hydroxybutanal to less toxic 1,3-butanediol, as suggested by its high activity towards this reaction (Table 1).

**Fig. 2:**
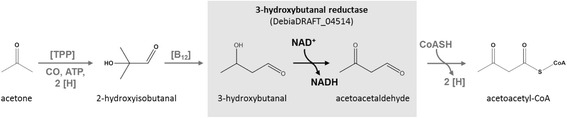
Illustration of a postulated pathway for acetone degradation in *Desulfococcus biacutus* with an attributed role of Debia-MDR as 3-hydroxybutanal dehydrogenase. In this hypothetical pathway, acetone would be carbonylated or formylated to a branched-chain aldehyde and then isomerized to linear 3-hydroxybutanal. The 3-hydroxybutanal would be oxidized to acetoacetaldehyde by the enzyme described in this study. Subsequently, acetoacetaldehyde could be converted to acetoacetyl-CoA (see also main text)

Third, when considering the abovementioned proposed pathway, and as illustrated in Fig. 2, Debia-MDR might also play a role in the oxidation of the proposed 3-hydroxybutanal intermediate to acetoacetaldehyde, as suggested by its high activity towards this reaction (Table 1). Moreover, the extremely reactive acetoacetaldehyde was previously shown (as dinitrophenylhydrazone adduct) to appear at low concentration in cell-free extracts of *D. biacutus* in the presence of acetone and CO [8]. Notably, the specific activity of Debia-MDR dehydrogenase for 3-hydroxybutanal oxidation to acetoacetaldehyde determined in vitro (83 nmol min^−1^ mg^−1^) is sufficient to explain the specific substrate turnover rate of *D. biacutus* during growth with acetone and sulfate (19 nmol min^−1^ mg^−1^) [34], though upstream and downstream processes may influence the rate of this reaction step in vivo.

Finally, in the context of the observed bifunctionality of Debia-MDR for both oxidation and reduction of 3-hydroxybutanal, it is tempting to speculate further whether the enzyme might play roles for both detoxifying, e.g., 3-hydroxybutanal to 1,3-butandiol and converting it to acetoacetyldehyde, dependent on the intracellular conditions: the latter reaction is catalyzed at lower rate, but may be facilitated if the subsequent enzyme in the pathway efficiently removes acetoacetaldehyde (an CoA-acylating aldehyde dehydrogenase [8, 9]). On the other hand, at times of 3-hydroxybutanal accumulation, it may reversibly be deposited as less toxic 1,3-butandiol (the equilibrium of this reaction is far on the side of the alcohol).

## Conclusion

Clearly, more work lies ahead to reveal the unusual acetone activation pathway in *D. biacutus*, which is hampered by, e.g., the absence of molecular genetic methods for this bacterium, the unavailability of proposed intermediates for conducting appropriate enzyme tests, and by the extremely labile, oxygen-sensitive enzyme activities. However, in this study, we report the first heterologous overproduction of a functional protein from *D. biacutus*, exhibiting an aldehyde/alcohol dehydrogenase activity for a broad range of short- and medium chain aldehydes and ketones in vitro. The enzyme appears to be involved in acetone degradation by this bacterium, and its recombinant production may represent an important step in the elucidation of the complete degradation pathway.

## Additional file

Additional file 1:
**Figure S1.** Determination of kinetic parameters of Debia-MDR with substrate acetone; **Table S1.** Specific activity of recombinant Debia-MDR at different pH values; **Table S2.** Specific activity of recombinant Debia-MDR at different reaction temperatures. (DOCX 44 kb)

## Abbreviations

ADH: Aldehyde dehydrogenase
IPTG: Isopropyl β-D-1-thiogalactopyranoside
MDR: Medium-chain dehydrogenase/reductase superfamily alcohol dehydrogenase
Ni-NTA: Nickel-nitrilotriacetic acid
PAGE: Polyacrylamide gel electrophoresis
