# Supplementary material for: Cloning, functional expression and characterization of a bifunctional 3-hydroxybutanal dehydrogenase /reductase involved in acetone metabolism by Desulfococcus biacutus
Source: BMC Microbiol. 2016 Nov 25;16:280. doi: 10.1186/s12866-016-0899-9 (PMC5123277; doi:10.1186/s12866-016-0899-9)
Supplement: Additional file 1: — Figure S1. Determination of kinetic parameters of Debia-MDR with substrate acetone; Table S1. Specific activity of recombinant Debia-MDR at different pH values; Table S2. Specific activity of recombinant Debia-MDR at different reaction temperatures. (DOCX 44 kb) [file 12866_2016_899_MOESM1_ESM.docx]

**Additional files**

Cloning, functional expression and characterization of a
bifunctional 3-hydroxybutanal dehydrogenase /reductase
involved in acetone metabolism by *Desulfococcus biacutus*

Jasmin Frey, Hendrik Rusche, David Schleheck and Bernhard Schink

Department of Biology, University of Konstanz, D-78457 Konstanz, Germany

Target journal: BMC Microbiology (Research article)

**Fig. S1 Determination of kinetic parameters of Debia-MDR with substrate acetone.**K_m_ value and V_max_ were calculated from triplicates with non-linear regression using SigmaPlot11 (Systat Software GmbH, Germany).

acetone concentration

**Table S1: Specific activity of recombinant Debia-MDR at different pH values.**Tested with 5 mM butanal and 0.1 mM NADH at 30 °C.

| pH | Specific activity (mU/mg protein) | % relative activity |
| --- | --- | --- |
| 6 | 71 ± 36 | 24 |
| 7.2 | 301 ± 24 | 100 |
| 8 | 370 ± 48 | 123 |
| 9 | 120 ± 35 | 40 |

**Table S2: Specific activity of recombinant Debia-MDR at different reaction temperatures.** Tested with 5 mM butanal and 0.1 mM NADH at pH 7.2.

| Temperature | Specific activity (mU mg^-1^ protein) | % relative activity |
| --- | --- | --- |
| 25 °C | 103 ± 21 | 34 |
| 30 °C | 301 ± 24 | 100 |
| 35 °C | 361 ± 45 | 120 |
| 37 °C | 474 ± 33 | 158 |
| 40 °C | 611 ± 80 | 203 |
| 45 °C | 656 ± 47 | 218 |
| 50 °C | 52 ± 4 | 17 |
